# Delivery Outcomes During the COVID-19 Pandemic as Reported in a Pregnancy Mobile App: Retrospective Cohort Study

**DOI:** 10.2196/27769

**Published:** 2021-10-04

**Authors:** Katie Noddin, Dani Bradley, Adam Wolfberg

**Affiliations:** 1 Ovia Health Boston, MA United States; 2 Cambridge Hospital Cambridge, MA United States

**Keywords:** digital health, COVID-19, maternal health, obstetrics, COVID, pandemic, pregnant women, birth, hospital, delivery, women's health, Cesarean sections

## Abstract

**Background:**

The COVID-19 pandemic has presented obstacles for providers and patients in the maternal health care setting, causing changes to many pregnant women’s birth plans, as well as abrupt changes in hospital labor and delivery policies and procedures. Few data exist on the effects of the COVID-19 pandemic on the maternal health care landscape at the national level in the United States.

**Objective:**

The aim of this study is to assess the incidence of key obstetrics outcomes (preterm delivery, Cesarean sections, and home births) and length of hospital stay during the COVID-19 pandemic as compared to the 6 months prior.

**Methods:**

We conducted a retrospective cohort study of women aged 18-44 years in the United States who delivered between October 1, 2019, and September 30, 2020, had singleton deliveries, and completed a birth report in the Ovia Pregnancy mobile app. Women were assigned to the prepandemic cohort if they delivered between October 2019 and March 2020, and the pandemic cohort if they delivered between April and September 2020. Gestational age at delivery, delivery method, delivery facility type, and length of hospital stay were compared.

**Results:**

A total of 304,023 birth reports were collected, with 152,832 (50.26%) in the prepandemic cohort and 151,191 (49.73%) in the pandemic cohort. Compared to the prepandemic cohort, principal findings indicate a 5.67% decrease in preterm delivery rates in the pandemic cohort (*P*<.001; odds ratio [OR] 0.94, 95% CI 0.91-0.96), a 30.0% increase in home birth rates (*P*<.001; OR 1.3, 95% CI 1.23-1.4), and a 7.81% decrease in the average hospital length of stay postdelivery (mean 2.48 days, SD 1.35). There were no overall changes in Cesarean section rates between cohorts, but differences were observed between age, race, and ethnicity subgroups.

**Conclusions:**

Results suggest a need for continuous monitoring of maternal health trends as the COVID-19 pandemic progresses and underline the important role of digital data collection, particularly during the pandemic.

## Introduction

The first confirmed case of COVID-19 in a pregnant woman in the United States was during the week of January 19, 2020. By March 8, there were over 100 confirmed cases in pregnant women per day, increasing to over 2000 cases per day by the first peak in early July [1]. By mid-to-late March 2020, the World Health Organization had declared COVID-19 a pandemic, and shortly thereafter states initiated stay-at-home orders, the Centers for Medicaid and Medicare Services expanded its coverage to include telehealth services, international travel was restricted, clinical trials were stalled, and the health care landscape was changed indefinitely [2]. During the following 16 months, and at the time of this writing, the COVID-19 pandemic presented novel obstacles for patients and providers in the maternal health care setting. For pregnant women, the risk of infection has been a source of fear and anxiety, causing many to rethink birth plans [3]. For hospitals, the virus has forced changes to labor and delivery policies and procedures, including increased restrictions on the number of allowed support persons and visitors, reduced intermediary locations for admitted patients, and expedited postpartum discharges [4].

Several studies have explored the effects of COVID-19 infection in pregnant women and on their birth outcomes, but there remains a lack of data (particularly at the national level) describing the effects of the pandemic itself—including infections and policy and lifestyle adjustments—on birth outcomes. Early studies on potential pandemic effects show decreases in preterm deliveries [5,6], as well as labor and delivery units with reductions in hospital length of stay [7]. The free Ovia Pregnancy (Ovia Health) mobile app, developed to help support women throughout their pregnancies, is uniquely positioned to address this gap by tracking real-time pregnancy and birth outcomes data on a national scale. Annually, the app serves approximately 3 million women and families across 50 states, with 60% of users logging in on iOS devices and 40% on Android.

Using user-reported data from the Ovia Pregnancy mobile app, we assessed key obstetrics outcomes throughout the COVID-19 pandemic and compared them to outcomes in the 6 months prior to the pandemic. This short paper focuses on the incidence of preterm delivery, Cesarean sections, and home births, as well as the length of hospital stays postdelivery during the first 6 months of the pandemic and the preceding 6 months.

## Methods

### Study Design

We conducted a retrospective cohort study of women aged 18-44 years residing in the United States who had singleton deliveries between October 1, 2019, and September 30, 2020, and completed a birth report in a pregnancy mobile app. The birth report collected delivery date, delivery method, delivery facility type, and hospital admission and discharge dates. We assigned women to the prepandemic cohort if they delivered between October 1, 2019, and March 30, 2020, and to the pandemic cohort if they delivered between April 1, 2020, and September 30, 2020. We compared gestational age at delivery, delivery method, delivery facility type, and hospital length of stay. Preterm delivery was defined as a baby born before 37 weeks of pregnancy. Delivery method options were vaginal, planned and unplanned Cesarean sections, and vaginal birth after Cesarean (VBAC). Delivery facility type options included hospital, birthing center, home birth, or other. Hospital length of stay was equal to the difference in days between hospital admission date and discharge date and was limited to those who reported stays ≤14 days. Demographic data were collected via Ovia Pregnancy app questions delivered to users as part of their app experience. With the exception of age, all demographic questions were optional.

### Statistical Analysis

All analyses were conducted in R Studio (version 1.3.959; R Foundation for Statistical Computing). Descriptive statistics were calculated using the *describeBy* function and unadjusted odds ratios for categorical variables were computed using the *odds.ratio* function. Proportions tests were conducted using the *prop.test* function. Means were compared using two-sample *t* tests. Relative change from prepandemic to pandemic was also calculated for all outcomes. This study was granted exemption by an independent review board (Advarra).

### Data Privacy

All of the data used in the study were collected from US resident users. All of the personal information collected by Ovia is processed in accordance with Ovia’s Privacy Policy [8] and applicable law.

## Results

### Sample Cohorts and Demographics

A total of 304,023 pregnant women in the United States between the ages of 18 and 44 years completed a birth report via the Ovia Pregnancy app and were thus eligible for the study. Among those, 152,832 (50.26%) women delivered between October 2019 and March 2020 and were assigned to the prepandemic cohort and 151,191 (49.73%) delivered between April 2020 and September 2020 and were assigned to the pandemic cohort. Women who reported their births represented 30.37% (prepandemic) and 31.10% (pandemic) of all women who used the app and also were expected to deliver during the respective time periods based on their logged last menstrual period date. The sample used in this study represents approximately 8.11% of annual births in the United States [9].

Among all users in the sample, 14.9% (n=45,530) completed questions about their race, 20.4% (n=61,886) completed questions about their education, 58.3% (n=177,359) completed questions about their employment status, and 21.7% (n=65,957) completed questions about their income. The majority identified as White (n=32,477, 71.33%), college-educated (n=23,085, 37.30%), and employed (n=131,420, 74.09%), and had annual household incomes over $100,000 (n=15,997, 24.25%). The average age at delivery was 28.31 years, and users in the pandemic cohort were, on average, slightly older. Demographic stratifications by cohort are shown in Table 1.

**Table 1:** Sample demographics prepandemic and during the pandemic.

Variables		Prepandemic (October 2019-March 2020; n=152,832)	Pandemic (April-September 2020; n=151,191)
Age at delivery (years), mean (SD)		28.2 (5.28)	28.5 (5.23)
**Age group at delivery (years), n (%)**			
	<20	5775 (3.78)	4848 (3.21)
	20-24	36,323 (23.77)	33,658 (22.26)
	25-29	48,026 (31.42)	47,258 (31.26)
	30-34	43,682 (28.58)	45, 687 (30.22)
	35-39	15,474 (10.78)	17,152 (11.34)
	40-44	2552 (1.67)	2588 (1.71)
**Race, n (%)**			
	White (non-Hispanic)	16,571 (71.04)	15,906 (71.63)
	Black (non-Hispanic)	1584 (6.79)	1467 (6.61)
	Asian American/Pacific Islander	415 (1.78)	372 (1.68)
	Hispanic/Latinx	1782 (7.64)	1704 (7.67)
	Multiracial	2973 (12.75)	2756 (12.41)
**Annual household income ($), n (%)**			
	<25,000	5370 (15.87)	4655 (14.49)
	25,000-50,000	8047 (23.78)	7436 (23.15)
	50,000-75,000	6311 (18.65)	6181 (19.24)
	75,000-100,000	6034 (17.83)	5926 (18.45)
	>100,000	8072 (23.86)	7925 (24.67)
**Completed education level, n (%)**			
	Some high school	917 (2.91)	900 (2.96)
	High school degree/equivalent	4123 (13.10)	3689 (12.13)
	Some college	7711 (24.50)	7331 (24.10)
	College degree	11,670 (37.08)	11,415 (37.53)
	Some postgraduate studies	1446 (4.59)	1463 (4.81)
	Postgraduate degree	5604 (17.81)	5617 (18.47)
**Employment status, n (%)**			
	Employed	65,619 (73.53)	65,801 (74.67)
	Not employed	23,619 (26.47)	22,320 (25.33)

### Preterm Delivery

**Figure 1:**
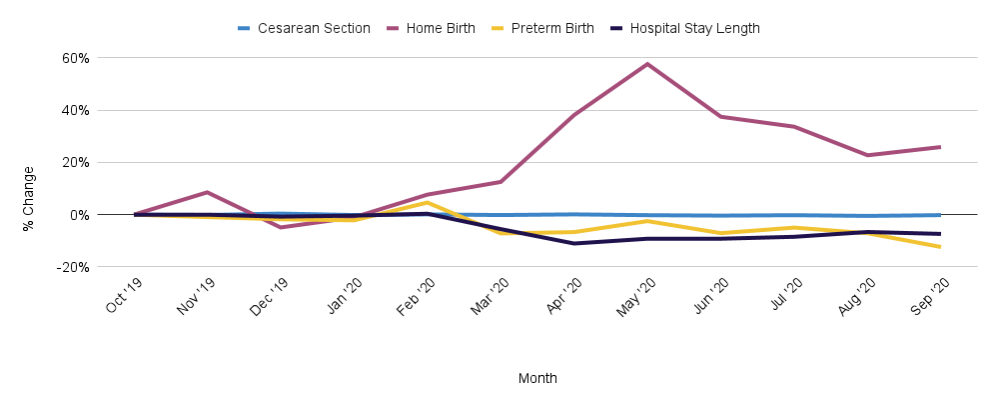
Relative change in reported birth outcomes by month, compared to reference period (October 2019).

A total of 272,686 (89.69%) users in the sample had valid gestational ages at delivery based on the last menstrual period date. Overall preterm delivery rates had a relative decrease of 5.67%, from 8.46% (n=11,192) in the prepandemic cohort to 7.98% (n=11,216) in the pandemic cohort (*P*<.001; odds ratio [OR] 0.94, 95% CI 0.91-0.96; Table 2). When compared to the reference period of October 2019, the overall greatest relative decrease in preterm deliveries was in September 2020 (Figure 1). Those aged 25-29 years had the greatest relative decrease in preterm delivery rates at 9.70%, from 8.36% (n=3422) in the prepandemic cohort to 7.55% (n=3286) in the pandemic cohort (*P*<.001; OR 0.90, 95% CI 0.85-0.94), followed by those aged 30-34 years, who had a 7.24% relative decrease, from 8.10% (n=3036) to 7.52% (n=3223; *P*=.002; OR 0.92, 95% CI 0.87-0.97; Table 3). Compared to other races and ethnicities, White non-Hispanic users had the greatest relative decrease in preterm deliveries at 6.28%, from 7.74% (n=1069) to 7.25% (n=1069; *P*<.001; OR 0.85, 95% CI 0.78-0.94; Table 4).

**Table 2:** Comparison of birth outcomes prepandemic and during the pandemic.

Birth outcomes		Value, n (%)		Relative change, %	*P* value	Odds ratio (95% CI)
		Prepandemic	During the pandemic			
Reported births, n (%)		152,832 (50.26)	151,191 (49.73)	–1.05	N/A^a^	N/A
**Gestational age at delivery, n (%)**						
	Full-term births	121,113 (91.54)	129,165 (92.01)	0.51	<.001^b^	0.94 (0.91-0.96)
	Preterm births (<37 weeks)	11,192 (8.46)	11,216 (7.98)	–5.67	<.001^b^	0.94 (0.91-0.96)
**Delivery method, n (%)**						
	Vaginal	103,808 (67.98)	102,717 (67.95)	–0.04	.79	1 (0.98-1.01)
	Cesarean section	46,923 (30.73)	46,381 (30.68)	–0.16	.79	0.99 (0.98-1.01)
	Vaginal birth after Cesarean	1981 (1.30)	2072 (1.37)	5.38	.29	0.96 (0.91-1.02)
**Delivery facility type, n (%)**						
	Hospital	141,267 (91.76)	141,173 (90.97)	–0.86	<.001^b^	0.90 (0.87-0.93)
	Birthing center	4812 (3.13)	4985 (3.21)	2.56	.11	1 (99.2-1.07)
	Home	1535 (1.00)	2019 (1.30)	30.00	<.001^b^	1.3 (1.23-1.4)
	Total out-of-hospital (birthing center + home)	6347 (4.12)	7004 (4.51)	9.47	<.001^b^	1.1 (1.06-1.14)
**Hospital stay length in days, mean (SD)**						
	All deliveries	2.69 (1.39)	2.48 (1.35)	–7.81	<.001^b^	N/A
	Vaginal + vaginal birth after Cesarean	2.42 (1.19)	2.24 (1.16)	–7.44	<.001^b^	N/A
	Cesarean section	3.46 (1.62)	3.17 (1.59)	–8.38	<.001^b^	N/A

^a^N/A: not applicable.

^b^5% statistical significance cutoff.

**Table 3:** Comparison of birth outcomes (preterm or full-term) prepandemic and during the pandemic by age group at delivery.

Age group (years), n (%)	Prepandemic, n (%)		Pandemic, n (%)		Relative change, %	*P* value	Odds ratio (95% CI)
	Preterm	Full-term	Preterm	Full-term	Preterm		
<20	474 (9.25)	4648 (90.75)	411 (9.27)	4022 (90.73)	0.19	1	1 (0.87-1.15)
20-24	2587 (8.15)	29,153 (91.85)	2572 (8.36)	28,190 (91.64)	2.58	.35	1.02 (0.97-1.08)
25-29	3422 (8.36)	37,498 (91.64)	3286 (7.55)	40,231 (92.45)	–9.70	<.001^a^	0.90 (0.85-0.94)
30-34	3036 (8.10)	34,427 (91.90)	3223 (7.52)	39,653 (92.48)	–7.24	.002^a^	0.92 (0.87-0.97)
35-39	1363 (9.29)	13,307 (90.71)	1424 (8.74)	14,873 (91.26)	–5.95	.09	0.93 (0.86-1.01)
40-44	310 (12.97)	2080 (87.03)	300 (12.02)	2196 (87.98)	–7.34	.34	0.91 (0.77-1.09)

^a^5% statistical significance cutoff.

**Table 4:** Comparison of birth outcomes (preterm versus full-term) prepandemic and during the pandemic by race and ethnicity.

Race and ethnicity	Prepandemic, n (%)		Pandemic, n (%)		Relative change, %	*P* value	Odds ratio (95% CI)
	Preterm	Full-term	Preterm	Full-term	Preterm		
White (non-Hispanic)	1069 (7.74)	12,751 (92.26)	1069 (7.25)	13,677 (92.75)	–6.28	<.001^a^	0.85 (0.78-0.94)
Black (non-Hispanic)	160 (11.28)	1259 (88.72)	170 (12.59)	1180 (87.41)	0.13	.31	1.13 (0.90-1.43)
Asian American/Pacific Islander	30 (8.19)	336 (91.81)	33 (9.42)	317 (90.58)	15.03	.65	1.16 (0.69-1.97)
Hispanic/Latinx	154 (9.91)	1399 (90.09)	146 (9.24)	1433 (90.76)	–6.76	.85	1.03 (0.81-1.30)
Multiracial	241 (9.62)	2262 (90.38)	237 (9.47)	2265 (90.53)	–1.62	.88	0.98 (0.81-1.18)

^a^5% statistical significance cutoff.

### Cesarean Sections

Among the total sample, 303,882 (99.9%) users completed the delivery method field of the app’s birth report form. Overall Cesarean section rates did not change significantly in the pandemic cohort compared to the prepandemic cohort (Table 2); however, there was a 11.68% relative increase in Cesareans in users under 20 years old, from 19.13% (n=1105) to 21.37% (n=1036; *P*=.004; OR 1.15, 95% CI 1.04-1.26; Table 5). Conversely, those aged 30-34 years had a 2.11% relative decrease, from 33.39% (n=14,539) to 32.68% (n=14,930; *P*=.02; OR 0.96, 95% CI 0.94-0.99; Table 5). Compared to other races and ethnicities, Black non-Hispanic users had the greatest difference in Cesarean rates with a 10.22% relative increase, from 36.48% (n=578) to 40.21% (n=590; *P*=.03; OR 1.17, 95% CI 1.01-1.35; Table 6).

**Table 5:** Comparison of birth outcomes (delivery method) prepandemic and during the pandemic by age group at delivery.

Age group (years), n (%)	Prepandemic, n (%)		Pandemic, n (%)		Relative change, %	*P* value	Odds ratio (95% CI)
	Cesarean section	Vaginal/vaginal birth after Cesarean	Cesarean section	Vaginal/vaginal birth after Cesarean	Cesarean section		
<20	1105 (19.13)	4670 (80.87)	1036 (21.37)	3812 (78.63)	11.68	.004^a^	1.15 (1.04-1.26)
20-24	9124 (25.13)	27,187 (74.87)	8598 (25.55)	25,059 (74.45)	1.67	.20	1.02 (0.98-1.06)
25-29	14,103 (29.38)	33,903 (70.62)	13,664 (28.92)	33,589 (71.08)	–1.57	.12	0.97 (0.95-1.00)
30-34	14,563 (33.39)	29,052 (66.61)	14,930 (32.68)	30,749 (67.32)	–2.11	.025^a^	0.96 (0.94-0.99)
35-39	6774 (41.16)	9684 (58.84)	6899 (40.24)	10,247 (59.76)	–2.24	.08	0.96 (0.92-1.00)
40-44	1254 (49.23)	1293 (50.77)	1254 (48.47)	1333 (51.53)	–1.55	.60	0.97 (0.86-1.08)

^a^5% statistical significance cutoff.

**Table 6:** Comparison of birth outcomes (delivery method) prepandemic and during the pandemic by race and ethnicity.

Race and ethnicity	Prepandemic, n (%)		Pandemic, n (%)		Relative change, %	*P* value	Odds ratio (95% CI)
	Cesarean section	Vaginal/vaginal birth after Cesarean	Cesarean section	Vaginal/vaginal birth after Cesarean	Cesarean section		
White (non-Hispanic)	5070 (30.60)	11,494 (69.40)	4849 (30.49)	11,053 (69.51)	–0.38	.83	0.99 (0.94-1.04)
Black (non-Hispanic)	578 (36.48)	1006 (63.52)	590 (40.21)	877 (59.79)	10.22	.03^a^	1.17 (1.01-1.35)
Asian American/Pacific Islander	133 (32.04)	282 (67.96)	124 (33.33)	248 (66.67)	4.01	.75	1.06 (0.78-1.42)
Hispanic/Latinx	578 (32.45)	1203 (67.55)	550 (32.27)	1154 (67.73)	–0.54	.94	0.99 (0.86-1.14)
Multiracial	928 (31.21)	2045 (68.79)	839 (30.44)	1917 (69.56)	–2.47	.54	0.96 (0.86-1.07)

^a^5% statistical significance cutoff.

### Out-of-Hospital Births

Among the sample, 295,791 (97.29%) users provided their birth facility type in the app. Total out-of-hospital birth rates increased by 9.47%, from 4.12% (n=6347) to 4.51% (n=7004; *P*<.001; OR 1.1, 95% CI 1.06-1.14). When assessing home birth rates alone, there was a 30.00% relative increase in pandemic rates from 1.00% (n=1535) to 1.30% (n=2019; *P*<.001; OR 1.3, 95% CI 1.23-1.40; Table 2). The overall relative increase in home birth rates peaked in May 2020 and remained consistently high through the end of the study period (Figure 1). Users aged 35-39 years had the greatest change in home birth rates at 37.18%, increasing to 1.60% (n=270) from 1.17% (n=186; Table 7). There were no statistically significant differences when stratifying by race and ethnicity (Table 8).

**Table 7:** Comparison of birth outcomes (delivery location) prepandemic and during the pandemic by age group at delivery.

Age group (years), n (%)	Prepandemic, n (%)		Pandemic, n (%)		Relative change, %	*P* value	Odds ratio (95% CI)
	Home birth	Other	Home birth	Other	Home birth		
<20	33 (0.58)	5621 (99.42)	31 (0.65)	4756 (99.35)	10.95	.77	1.11 (0.67-1.82)
20-24	291 (0.83)	34,892 (99.17)	338 (1.02)	32,653 (98.98)	23.87	.007^a^	1.24 (1.06-1.45)
25-29	509 (1.10)	45,885 (98.90)	669 (1.45)	45,587 (98.55)	31.83	<.001^a^	1.32 (1.18-1.49)
30-34	486 (1.16)	41,508 (98.84)	672 (1.50)	44,087 (98.50)	29.73	<.001^a^	1.30 (1.16-1.46)
35-39	186 (1.17)	15,731 (98.83)	270 (1.60)	16,573 (98.40)	37.18	<.001^a^	1.37 (1.14-1.66)
40-44	30 (1.21)	2442 (98.79)	39 (1.53)	2502 (98.47)	26.47	.39	1.26 (0.78-2.06)

^a^5% statistical significance cutoff.

**Table 8:** Comparison of birth outcomes (delivery location) prepandemic and during the pandemic by race and ethnicity.

Race and ethnicity	Prepandemic, n (%)		Pandemic, n (%)		Relative change, %	*P* value	Odds ratio (95% CI)
	Home birth	Other	Home birth	Other	Home birth		
White (non-Hispanic)	249 (1.54)	15,824 (98.46)	281 (1.80)	15,319 (98.20)	16.27	.08	1.16 (0.98-1.34)
Black (non-Hispanic)	14 (0.90)	1527 (99.10)	20 (1.38)	1422 (98.62)	52.66	.29	1.52 (0.77-3.11)
Asian American/Pacific Islander	3 (0.75)	396 (99.25)	4 (1.08)	364 (98.92)	44.57	.71	1.44 (0.23-9.96)
Hispanic/Latinx	10 (0.58)	1713 (99.42)	12 (0.71)	1665 (99.29)	23.29	.78	1.23 (0.52-2.95)
Multiracial	52 (1.80)	2822 (98.20)	69 (2.56)	2617 (97.44)	41.98	.06	1.42 (0.99-2.06)

### Hospital Length of Stay

A total of 122,613 (40.33%) users who delivered in a hospital provided their admittance and discharge dates in the app. Average hospital length of stay decreased by 7.81% in the pandemic cohort (mean 2.48 days, SD 1.35) as compared to the prepandemic cohort (mean 2.69, SD 1.39; Table 2). The largest overall decrease in hospital length of stay was in April, compared to the reference period of October 2019 (Figure 1). Results were similar when stratified by birth method; mean hospital length of stay decreased by 8.38% for Cesarean sections and mean length of stay decreased by 7.44% for vaginal deliveries (Table 2). Users aged 40-44 years had the greatest decrease in mean hospital length of stay, both overall and for Cesarean deliveries, at 10.06% (mean 2.77, SD 1.65) and 14.84% (mean 3.27, SD 1.78), respectively (Table 9). Among vaginal deliveries, women aged 30-34 years had the greatest decrease in length of stay at 7.92%. Hospital length of stay decreases persisted across race and ethnicity groups. For all deliveries, multiracial users had the greatest decrease in length of stay at 11.41% (Table 10). For Cesarean sections, Asian American/Pacific Islander users had a 9.8% decrease in length of stay. For vaginal and VBAC births, Hispanic and Latinx users had the greatest decrease in length of stay at 7.92%.

**Table 9:** Comparison of hospital length of stay after delivery prepandemic and during the pandemic, by age group at delivery.

Age groups by delivery type		Hospital length of stay (days), mean (SD)		Relative change, %	*P* value
		Prepandemic	Pandemic		
**Age groups for all deliveries (years)**					
	<20	2.73 (1.32)	2.56 (1.24)	–6.23	<.001^a^
	20-24	2.65 (1.33)	2.48(1.33)	–6.42	<.001^a^
	25-29	2.66 (1.4)	2.66 (1.32)	0.38	<.001^a^
	30-34	2.7 (1.41)	2.48 (1.36)	–6.77	<.001^a^
	35-39	2.84 (1.51)	2.58 (1.43)	–9.15	<.001^a^
	40-44	3.08 (1.62)	2.77 (1.65)	–10.06	<.001^a^
**Age groups for Cesarean sections (years)**					
	<20	3.47 (1.52)	3.3 (1.42)	–4.90	.05
	20-24	3.42 (1.57)	3.19 (1.62)	–6.73	<.001^a^
	25-29	3.47 (1.67)	3.13 (1.52)	–9.80	<.001^a^
	30-34	3.44 (1.59)	3.18 (1.63)	–7.56	<.001^a^
	35-39	3.52 (1.60)	3.19 (1.58)	–9.38	<.001^a^
	40-44	3.84 (1.83)	3.27 (1.78)	–14.84	<.001^a^
**Age groups for vaginal + vaginal birth after Cesarean deliveries (years)**					
	<20	2.57 (1.22)	2.38 (1.13)	–7.39	<.001^a^
	20-24	2.44 (1.16)	2.28 (1.16)	–6.56	<.001^a^
	25-29	2.38 (1.18)	2.21 (1.15)	–7.14	<.001^a^
	30-34	2.40 (1.20)	2.21 (1.13)	–7.92	<.001^a^
	35-39	2.46 (1.32)	2.27 (1.23)	–7.72	<.001^a^
	40-44	2.50 (1.15)	2.41 (1.44)	–3.60	.26

^a^5% statistical significance cutoff.

**Table 10:** Comparison of hospital length of stay prepandemic and during the pandemic, by race and ethnicity.

Race/ethnicity by delivery type		Hospital length of stay (days), mean (SD)			Relative change, %	*P* value
		Prepandemic		Pandemic		
**Race/ethnicity for all deliveries**						
	White (non-Hispanic)	2.58 (1.36)	2.32 (1.24)		–10.47	<.001^a^
	Black (non-Hispanic)	2.84 (1.45)	2.57 (1.43)		–9.51	<.001^a^
	Asian American/Pacific Islander	2.74 (1.57)	2.46 (1.15)		–10.22	<.001^a^
	Hispanic/Latinx	2.44 (1.21)	2.33 (1.29)		–4.51	.07
	Multiracial	2.63 (1.41)	2.33 (1.24)		–11.41	<.001^a^
**Race/ethnicity for Cesarean sections**						
	White (non-Hispanic)	3.47 (1.52)	3.3 (1.42)		–4.90	.05
	Black (non-Hispanic)	3.42 (1.57)	3.19 (1.62)		–6.73	<.001^a^
	Asian American/Pacific Islander	3.47 (1.67)	3.13 (1.52)		–9.80	<.001^a^
	Hispanic/Latinx	3.44 (1.59)	3.18 (1.63)		–7.56	<.001^a^
	Multiracial	3.52 (1.6)	3.19 (1.58)		–9.38	<.001^a^
**Race/ethnicity for vaginal + vaginal birth after Cesarean deliveries**						
	White (non-Hispanic)	2.57 (1.22)	2.38 (1.13)		–7.39	<.001^a^
	Black (non-Hispanic)	2.44 (1.16)	2.28 (1.16)		–6.56	<.001^a^
	Asian American/Pacific Islander	2.38 (1.18)	2.21 (1.15)		–7.14	<.001^a^
	Hispanic/Latinx	2.4 (1.2)	2.21 (1.13)		–7.92	<.001^a^
	Multiracial	2.46 (1.32)	2.27 (1.23)		–7.72	<.001^a^

^a^5% statistical significance cutoff.

## Discussion

### Principal Findings

This paper describes key birth outcomes during the COVID-19 pandemic. Our results indicate a decline in preterm births, a contrast to recent trends in the United States reflecting data from nonpandemic years [9,10]. These results were most prominent among those aged 25-29 years and 30-34 years, and among White users. The overall declines align with other reports indicating COVID-19–related decreases in preterm deliveries, many of which have suggested several plausible reasons for the decline, including less exposure to infection and other consequences of physical distancing, mask wearing, increased attention to health and exercise, and possible reduction in antenatal surveillance that might lead to medical interventions and early delivery [5,6]. As these studies also suggest, more in-depth research is needed to test the plausibility of any one hypothesis.

Overall results indicated no change in Cesarean section rates between the two cohorts, but age-specific results showed increases in Cesarean section rates among those under 20 years and decreases in those aged 30-34 years. When comparing race and ethnicity, Black non-Hispanic users had a significant increase in Cesareans compared to all other race groups. Special attention and further research should be conducted to address age-specific differences, as well as social determinants of health that disproportionately affect Black pregnant women, particularly during the COVID-19 pandemic.

We also found a significant increase in home births in just 6 months, compared to national reports indicating no change in home birth rates between 2018 and 2019 [9]. This change was especially apparent in users aged 35-39 years. It is important that providers be diligent in informing patients and providing appropriate resources about home birth risks, as planned home births are associated with poorer outcomes for most of the population, as compared to hospital births [11].

Our study also shows a decreased average length of stay after delivery among those who delivered in a hospital, particularly among those aged 40-44 years and those who are multiracial. Reduced hospital length of stay has both positive and negative implications: decreased hospital stay length could lead to increased readmission rates and costs, and poorer postpartum and neonatal outcomes [12]. Conversely, early discharge may reduce SARS-CoV-2 exposure with limited adverse consequences in low-risk patients [7].

### Limitations

Our study is limited in that those who choose to report the details of their deliveries in an app may differ from those who do not. We are also reliant on user-reported data, which we recognize can present additional biases. Relatedly, while we do present some demographic data in this paper, we are largely restrained by demographic data completeness for this population, as most demographic fields in the Ovia Pregnancy app are not required or collected in the sign-up process. As such, sample sizes were limited when performing stratified analyses, and in-app questions, such as household income, education level, and employment status may have been completed and unchanged outside of the study time period.

We also know that SARS-CoV-2 infection may play a significant role in the birth outcomes described here [13], and we are limited in that the Ovia Pregnancy app does not collect specific COVID-19 infection data.

### Conclusions

As the pandemic progresses, continuous monitoring of these trends and others is necessary to evaluate long-term effects on birth outcomes. The use of digital data collection is paramount to monitoring these trends in real time, particularly during a time when there are increased limitations regarding access to care.

## Abbreviations

OR: odds ratio
VBAC: vaginal birth after Cesarean
